# Impulsive Action and Impulsive Choice Are Differentially Associated With Gene Expression Variations of the GABA_A_ Receptor Alfa 1 Subunit and the CB_1_ Receptor in the Lateral and Medial Orbitofrontal Cortices

**DOI:** 10.3389/fnbeh.2019.00022

**Published:** 2019-02-20

**Authors:** Marcos Ucha, David Roura-Martínez, Ana Contreras, Sheyla Pinto-Rivero, Javier Orihuel, Emilio Ambrosio, Alejandro Higuera-Matas

**Affiliations:** Department of Psychobiology, School of Psychology, National University for Distance Education (UNED), Madrid, Spain

**Keywords:** impulsivity, orbitofrontal cortex, delay-discounting, two-choice serial reaction time task, inhibitory control, ionotropic receptors, GABA, endocannabinoid system

## Abstract

The orbitofrontal cortex (OFC) is a key brain region for decision-making, action control and impulsivity. Quite notably, previous research has identified a double dissociation regarding the role of this cortical territory in impulsive choice. While medial orbitofrontal lesions increase preference for a large but delayed reward, lateral orbitofrontal lesions have the opposite effect. However, there are no data regarding this anatomical dissociation in impulsive action. The neurochemical basis of impulsivity is still being elucidated, however, in recent years a role for the endocannabinoids and the related glutamatergic and GABAergic neurotransmitter systems has been suggested. Here, we submitted male Wistar rats to a delay-discounting task (DDT) or a two-choice serial reaction time task (2-CSRTT) and classified them as high impulsive or low impulsive in either task using cluster analysis. We then examined the gene expression of several elements of the endocannabinoid system or different subunits of certain glutamatergic or GABAergic ionotropic receptors (AMPA, NMDA, or GABA_A_) in the lateral or medial divisions of their orbitofrontal cortices. Our results confirm, at the gene expression level, the dissociation in the participation of the medial, and lateral divisions of the orbitofrontal cortex in impulsivity. While in the 2-CSRTT (inhibitory control) we found that high impulsive animals exhibited lower gene expression levels of the α1 GABA_A_ receptor subunit in the lateral OFC, no such differences were evident in the medial OFC. When we analyzed DDT performance, we found that high impulsive animals displayed lower levels of CB_1_ gene expression in the medial but not in the lateral OFC. We propose that GABAergic dynamics in the lateral OFC might contribute to the inhibitory control mechanisms that are altered in impulsive behavior while endocannabinoid receptor gene transcription in the medial OFC may subserve the delay-discounting processes that participate in certain types of impulsiveness.

## Introduction

Understanding the mechanisms behind the control of behavior is one of the biggest challenges of modern Neuroscience. The natural tendency to make rapid decisions without foresight is a multifaceted trait commonly known as impulsivity. The capacity to make rapid decisions and act quickly without hesitation can be beneficial in many situations. However, when this tendency becomes extreme it can be detrimental and symptomatic of several psychopathological conditions such as attention deficit hyperactivity disorder or substance abuse (Dalley and Robbins, 2017).

During the last decades, researchers have explored different approaches to objectively measure impulsivity in humans and other mammals. There is an ample variety of tests based on decision making (such as delay and probability discounting tasks) and tests based on inhibiting motor actions [such as the five-choice serial reaction time task (5-CSRTT) or go-no go tasks]. Considering the last decade of research and on the grounds of the neuroanatomical circuits essential to each test, impulsiveness is categorized into “waiting impulsivity” [measured with the delay-discounting task (DDT) and the 5-CSRTT], “stopping impulsivity” or the difficulty to stop an already initiated action (go/no-go tasks) and the preference for uncertain but bigger outcomes, known as “risky impulsivity” (probability discounting tasks). Although all these kinds of impulsivity share some common neural mechanisms they also rely on independent pathways (for an excellent review read Dalley and Robbins, 2017).

Waiting impulsivity is usually assessed using delay-discounting or choice reaction time-based tasks. Nevertheless, these tasks could actually be assessing distinct subtypes of waiting impulsivity as they rely on subtly different neural mechanisms. For example, although both tasks are mediated by the nucleus accumbens (NAcc), the capacity of delaying gratification is more dependent on the core, while the inhibition of premature responses relies on the integrity of the shell (Basar et al., 2010). In addition, both subtypes of waiting impulsivity predict different aspects of drug addiction (Belin et al., 2008; Diergaarde et al., 2008).

The OFC has long been associated with several functions related to decision making (Wallis, 2007), including impulsivity (Chudasama et al., 2003; Berlin et al., 2004), but the concrete role of this area remains elusive. This elusiveness could be a consequence of the functional dissociation of the lateral and medial OFC shown both in humans (Elliott et al., 2000; Sescousse et al., 2010) and primates (Noonan et al., 2010). In rodents, the study of Mar et al. (2011) revealed a similar functional dissociation between the lOFC and the mOFC. The lesions in the lOFC elicited an increase in waiting impulsivity in a DDT whereas lesions of the mOFC caused the opposite effect. It may be tempting to speculate that this orbitofrontal dissociation could be related to the aforementioned segregation of functions between the core and shell of the NAcc, however, it seems that in the rat (contrary to the monkey) the NAcc is almost devoid of proper orbitofrontal connections [only the lateral portions of the shell receive some projections from the lOFC (Schilman et al., 2008)].

In this study, we set out to assess whether the expression of genes related to glutamatergic, GABAergic or cannabinoid neurotransmission in the lOFC or mOFC was related to the two varieties of waiting impulsivity that are captured by the DDT or the two choice serial reaction time task (2-CSRTT).

Most of the previous studies regarding impulsivity and the OFC have focused on other neurotransmitters such as dopamine and serotonin (Winstanley et al., 2006; Dalley et al., 2008). We chose to study the gene expression of several subunits of glutamatergic and GABAergic ionotropic receptors because of their direct relationship with the excitation or inhibition status of the region where they are being expressed and because little is still known about their roles in impulsivity. In addition, we have assessed endocannabinoid related gene expression because the endocannabinoid system plays a key role in the modulation GABA and glutamate release from the presynaptic terminals.

## Materials and Methods

### Animals

Adult male Wistar rats (*n* = 36, 18 per experiment) (Charles River Laboratories) were housed in groups of 3 in a controlled facility with a temperature of 22 ± 2°C and relative humidity of 50% ± 10 on an inverted 12 h/12 h light/dark cycle (lights on at 8:00 pm). The rats weighted around 300 g at the beginning of the experiments and were kept at around 90–95% of their original weight by restricting their access to food (standard commercial rodent diet A04/A03: Panlab). They had *ad libitum* access to water through all the duration of the experiments. All the animals were maintained and handled according to European Union guidelines for the care of laboratory animals (EU Directive 2010/63/EU governing animal experimentation).

### Apparatus

The behavioral tests were performed using six operant conditioning chambers (*l* = 300 mm; *w* = 245 mm; *h* = 328 mm) (Med Associates). The front part of each box was equipped with two levers 14 cm apart and a pellet dispenser with a nose poke detector between them. There were also light cues above each lever, a house light close to the top of the boxes and a white noise generator. The chambers were controlled using the software MedPC by a computer connected to a compatible interface (Med Associates).

### Behavioral Tasks

#### Acquisition of Lever Press Response

All the rats received instrumental training sessions with food pellets (grain-based rodent tablet, Testdiet^TM^) and a light cue indicating the active lever on a fixed ratio 1 schedule. The sessions lasted 30 min and continued daily until the animals developed an acceptable lever press behavior (at least 30 lever presses), and then the same training was performed with the other lever (the order of the levers was counterbalanced across the conditioning chambers). Once the animals reached the criterion for both levers, they were trained with both active levers simultaneously (both cue lights on/both levers reward) until the Left/Right lever ratio was 1:1 ± 10%.

#### Behavioral Measurements of Impulsivity

##### Delay-discounting task

For the study of “impulsive choice,” we used an adaptation of the protocol of the DDT described by Mar and Robbins (2007). Each session lasted 100 min and consisted of five blocks of 12 trials each. Trials are presented every 100 s (i.e., 60 trials in 100 min). One of the levers (the “immediate lever”) initiated the delivery of one food pellet when pressed while the other (the “delayed lever”) delivered four of them. The immediate and delayed levers were in the same location (left or right) for each animal, but their position was counterbalanced between animals. The delay between lever press and the delivery of the reward was always 0 s for the immediate lever, whereas the delay associated to the delayed lever was increased across blocks in order to assess the tolerance to delay of the rats. The first two trials of each block were forced (i.e., only one lever was active and its corresponding cue light was illuminated). During the rest of the trials both levers were available, a fact that was signaled by the illuminated cue lights above each lever. Once a lever was pressed within the 10 s interval given, the cue lights were turned off and an inter-trial interval (ITI) commenced. If the rat failed to respond during the 10 s period, all lights were turned off, punishing the omitted response. During the first training sessions, both levers delivered a reward immediately, and these sessions continued until the rats showed a clear preference for the lever that delivered the large reward (> 90% choice). Once the criterion was met, the rats started the test sessions in which the delay of delivery for the delayed lever was increased with every block change (0, 5, 10, 20, and 40 s, respectively). At the end of each block, a tone cue was presented to mark the beginning of the next block. The choice ratio for each block was calculated by dividing the number of delayed responses in all the free-choice trials of the block (a maximum of 10 free choice trials per block) by the number of free choice trials completed. We used the average of the choice ratio during three consecutive blocks as a reliable estimate of choice behavior.

The sessions were repeated daily until the rats achieved a stable delay-discounting performance. Due to the variability of discounting curves between rats, the criterion for stability was defined by the average behavior of all the rats. We performed a two-way repeated measures ANOVA with the average choice ratios during two contiguous 3-sessions blocks as the BLOCK dependent variable and 3-SESSIONS and DELAY as within-subject factors. Stability was met when no significant effect of the 3 SESSION BLOCK was found but a significant effect of DELAY was observed. This was achieved after twenty sessions of delay-discounting training.

Waiting impulsivity was operationalized here by the k parameter, calculated by fitting the choice ratio of the last three sessions block to a non-linear exponential function (CR = e^-k(DELAY )^). The k parameter determines the rate of decay of the exponential function, i.e., the rate at which the lever choice changes from delayed to immediate across delays. Consequently, larger *k*-values indicate a faster rate of lever choice change and more impulsive behavior (Odum, 2011). There are other methods to compare the behavior of delay-discounting curves across groups or subjects, like the normalized area under the curve (AUC) (Myerson et al., 2001) or the AUC without normalization (Magnard et al., 2018). Similarly to the k parameter, these two metrics provide an index that is comparable between studies. In addition to computing the k parameter, we have also extracted both AUCs measures and tried to cluster the rats using the two indices. The main correlation of this study was preserved using the both AUCs. However, the groups resulting from the clustering process had very different sample sizes and were not considered in this study (see Supplementary Information).

##### Two choice serial reaction time task

The two choice serial reaction time task (2-CSRTT) used here is an adaptation of the popular five-choice serial reaction time task (Bari et al., 2008). The 2-CSRTT has been shown to be sensitive to an amphetamine challenge which increased premature responding in the task while leaving other parameters unaffected (Van Gaalen et al., 2009). This task was carried out in the same conditioning boxes described for the DDT.

The task started once the nose poke detector sensed an entry in the pellet dispenser. One of the stimulus lights was turned on for a variable period of time. If the lever under the light was pressed during the response interval time (CORRECT response), a pellet was delivered and, after an ITI, the next trial started. If the rat pressed the wrong lever (ERROR response), pressed a lever before any stimulus (PREMATURE response), or did not press any lever at all (OMISSION response), then the house light was turned off and rewards were not available during 5 s as a punishment. The sessions finished after 100 trials or 30 min, whichever came first. Once a rat completed one session with more than 75% of correct responses and less than 20% of omissions, the next phase of the experiment started. The experiment consisted of 12 training phases and a test phase. As the phases progressed, the stimulus duration and response interval time were shortened, while the ITI was extended [as detailed in the excellent description of the protocol by Bari et al. (2008)]. In the test phase, the ITI was drastically increased to 9 s to increase the number of premature responses and unmask the latent impulsivity trait. We used this variable (number of premature responses during the test phase) as a measure of the motor component of waiting impulsivity of each rat.

### Sample Processing

**FIGURE 1:**
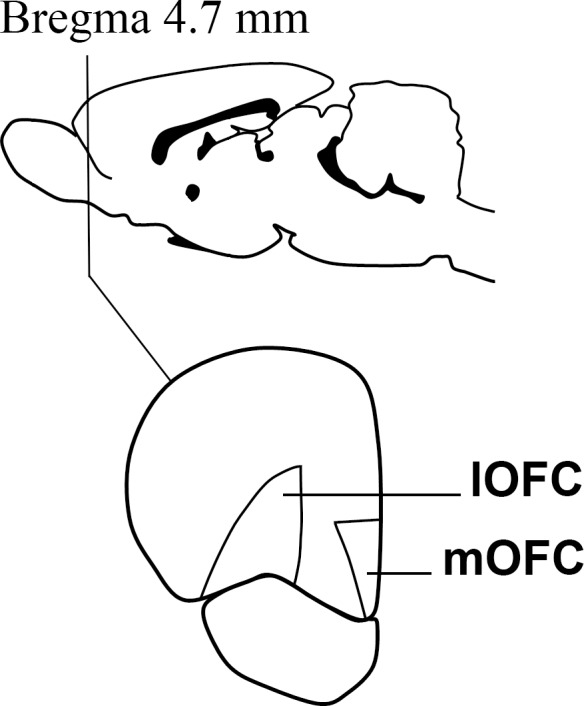
A cartoon depicting the approximate Bregma level at which dissections were made. The medial and lateral divisions of the orbitofrontal cortex were dissected out on ice with the help of the Paxinos and Watson atlas Paxinos and Watson (1998).

After the behavioral assessments, the animals of both experiments were left *ad libitum* in their home cages for one week, in order to prevent any effect of the behavioral tests on gene expression. Then, they were mildly anesthetized with isoflurane and euthanized by decapitation. Using tools and surfaces previously treated with RNAseZap (Ambion) to prevent RNA degradation, the brain was extracted and the mOFC and lOFC were dissected out of 1 mm slices obtained by using a brain matrix and the adequate equipment. The dissected areas are depicted in Figure 1. The samples were then snap frozen in dry ice and stored at -70°C for further processing. Five brains of the delay-discounting experiment were lost due to a faulty freezer.

### RT-qPCR

RNA was isolated using the commercial kit RNeasy Lipid Tissue Mini Kit (Qiagen). Samples were retrotranscribed using a commercial kit (Biorad iScript^TM^ cDNA Synthesis Kit). PCR assays were performed on a real-time PCR detection system (CFX9600, Bio-Rad) with an SSO Advanced SYBR mix (Bio-Rad) using the primers indicated in Supplementary Table S1. We assessed the expression of subunits of the NMDA glutamatergic receptor (R1 and 2A), AMPA receptor (GluA1 and GluA2), GABA_A_ receptor (alpha 1, alpha 2, delta, and gamma 2) an of elements of the endocannabinoid system (the CB_1_ receptor, the anandamide synthesis enzyme NAPE-PLD, the anandamide-degrading enzyme FAAH, the 2-arachidonoyl glycerol (2-AG) synthesis enzyme diacylglycerol lipase and the 2-AG degrading enzyme monoacylglycerol lipase). The relative expression of the target genes was calculated according to Pfaffl (2001), using *Gapdh* as a reference gene and the reaction efficiencies were obtained using LinRegPCR software (Ruijter et al., 2009).

### Statistical Analyses

The animals were classified according to their impulsivity using hierarchical cluster analysis with Ward’s method. Although other approaches, like a quartile categorization, could be applied to isolate extreme sub-populations in our sample, we were interested in studying the whole population so that we could compare these results with those obtained in the correlational analysis (which must include the whole behavioral and neurochemical continuum of the entire population). We also refrained from using a quartile approach because doing so would incur in loss of power due to resulting smaller sample size.

We analyzed the differences in the behavior of the clustered groups with a two-way repeated measures linear mixed models approach with either lever preference (for DDT) or premature responses (for 2-CSRTT) as the dependent variable, CLUSTER as the between-subject factor and DELAY or SESSION as the within-subject factor. We also used Student *t*-tests to test if the averages for k or the premature responses during the day of the test were significantly different between the clustered groups. Subsequently, we checked for statistical differences in gene expression between both groups using either the Student’s *t*-test for the homocedastic and normal data or Mann Whitney’s U when the parametric assumptions were not met. We applied a false discovery rate (FDR) correction using the Benjamini-Hochberg procedure with an FDR level of 0.1. We report Cohen’s *d* as the effect size estimator for parametric and r for non-parametric data. All the uncorrected *p*-values are available in the Supplementary Materials. Finally, we measured the relationship between the expression of the genes which were found to have differential expression between groups and either measure of impulsivity using Pearson’s r when the populations of both variables were normally distributed and Kendall’s τ for the non-parametric data.

All the statistical analyses were performed using SPSS 24 (IBM) or InVivoStat (Bate and Clark, 2011) and the level of significance was set to α = 0.05. All the graphs were designed using the PRISM 6 software (GraphPad Software, Inc.) or Photoshop (Adobe Systems Inc.).

## Results

### Delay-Discounting

**FIGURE 2:**
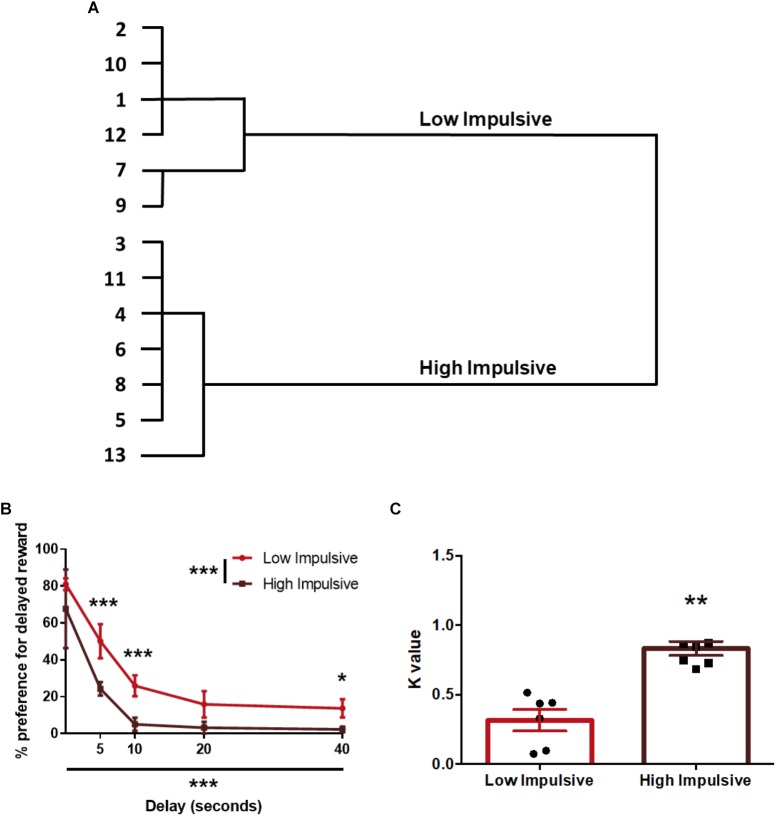
Population segregation according to performance in the delay-discounting task. **(A)** Cluster analysis dendrogram showing the grouping of rats in high impulsive and low impulsive populations. Numbers correspond to the ID of each rat according to our numbering system for this experiment. **(B)** Delay-discounting curves of high and low impulsive rats. ^∗^*p* < 0.05, ^∗∗^*p* < 0.01, and ^∗∗∗^*p* < 0.001 as compared to the low impulsive group. The main GROUP and DELAY effects are represented by the asterisks in the legend and in the horizontal axis. **(C)** *k-*value of high impulsive and low impulsive animals. ^∗∗^*p* < 0.01 as compared to the low impulsive group. Line and bar graphs represent the mean ± standard error of the mean. Symbols in bar graphs represent individual data points from each rat.

Regarding impulsivity measured with the DDT, we used the *k*-values of the animals to segregate them in two groups: 7 rats were assigned to the High Impulsive (HI-DD) group and 6 to the Low Impulsive (LI-DD) group (Figure 2A,B). As expected, HI-DD rats showed steeper discounting curves than LI-DD animals (significant CLUSTER^∗^DELAY interaction (*F*_4,44_ = 7.48; *p* < 0.001), significant effect of the CLUSTER factor (*F*_1,11_ = 12.57; *p* < 0.01) and significant DELAY factor (*F*_4,44_ = 51.56 *p* < 0.0001). We also compared the average *k*-value of both groups and verified that they differed significantly (*t*_11_ = -5.77; *p* < 0.001; *d* = -3.16; Figure 2C).

After the FDR correction, we found that the rats of the HI-DD group expressed higher levels of *Cnr1* in the mOFC than the rats of the LI-DD group (*t*_8_ = -4.13; *p* < 0.01; *d* = -2.71; Figure 4A). We also found a significant positive correlation between k and the expression of *Cnr1* in the mOFC (*r* = 0.77; *p* < 0.01 uncorrected). Accordingly, the animals that expressed higher levels of expression of these genes displayed higher impulsivity in this task (Figure 4B). There were no differences in *Cnr1* gene expression between HI-DD and LI-DD in the lOFC (Figure 4C) or in the *Gabra1* gene expression in either territory of the OFC (Figure 5D,E).

### Two-Choice Serial Reaction Time

**FIGURE 3:**
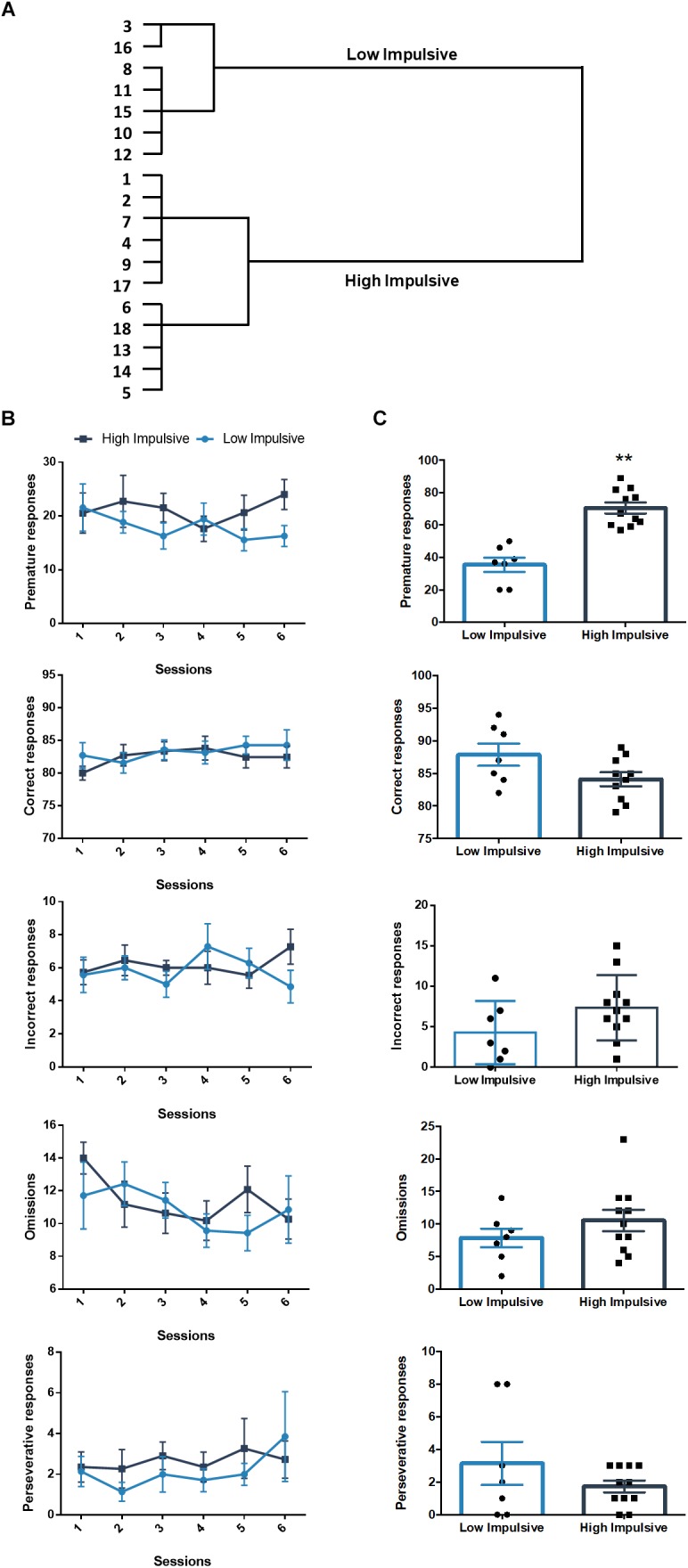
Population segregation according to performance in the 2-CSRTT. **(A)** Cluster analysis dendrogram showing the grouping of rats in high impulsive and low impulsive populations. Numbers correspond to the ID of each rat according to our numbering system for this experiment. These numbers represent different rats from those used in the DDT experiment. **(B)** Performance in the 2-CSRTT during the last six sessions, prior to the test day. There were no differences between both groups in either the premature, correct, incorrect, omitted or premature responses (Table 1). **(C)** Performance on the days of the test (ITI = 9 s). ^∗∗^*p* < 0.01 as compared to the low impulsive group. Line and bar graphs represent the mean ± standard error of the mean. Symbols in bar graphs represent individual data points from each rat.

We also sorted another set of rats that performed the 2-CSRTT according to their premature responses in the long-ITI test day, they clustered in two groups: a high impulsive group of 11 rats (HI-2C) and a low impulsive group of 7 rats (LI-2C) (Figure 3A). The repeated measures linear mixed model analysis revealed no differences between both groups in either the premature, correct, incorrect, omitted or premature responses (Table 1 and Figure 3B). During the test, no differences were found between both groups in the number of omissions, incorrect or perseverative responses but the number of premature responses during the test was significantly different between both groups (*t*_16_ = -6.385; *p* < 0.001; *d* = -3.07; Figure 3C).

**Table 1:** Results of the two-way repeated measures linear mixed model of the six last training sessions of the 2-CSRTT.

Responses	Cluster	Session	Cluster^∗^Session
Premature	*F*(1,16) = 0.23; *p* = 0.64	*F*(5,80) = 0.87; *p* = 0.5	*F*(5,80) = 0.65; *p* = 0.66
Correct	*F*(1,16) = 0.23; *p* = 0.64	*F*(5,80) = 0.87; *p* = 0.51	*F*(5,80) = 0.65; *p* = 0.66
Incorrect	*F*(1,16) = 0.12; *p* = 0.73	*F*(5,80) = 0.67; *p* = 0.64	*F*(5,80) = 1.7; *p* = 0.14
Omissions	*F*(1,16) = 0.19; *p* = 0.67	*F*(5,80) = 1.39; *p* = 0.24	*F*(5,80) = 0.87; *p* = 0.5
Perseverative	*F*(1,16) = 0.26; *p* = 0.62	*F*(5,80) = 0.85; *p* = 0.52	*F*(5,80) = 0.56; *p* = 0.73


**FIGURE 4:**
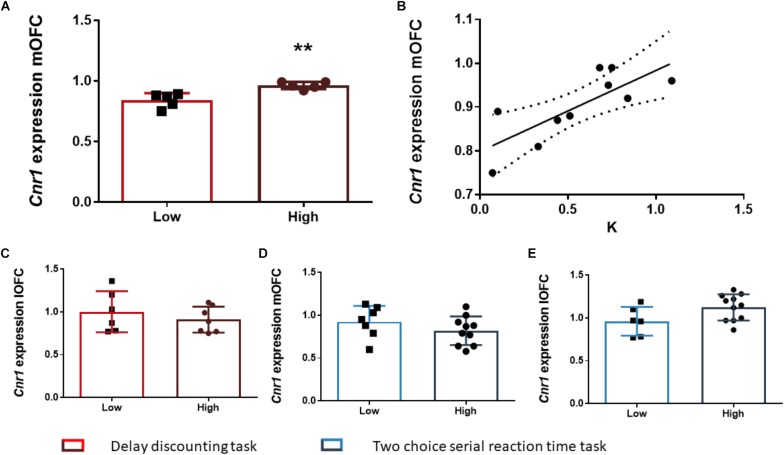
The relationship between *Cnr1* gene expression and impulsive behavior. **(A)** Rats classified as high impulsive according to their delay-discounting showed a significantly higher expression of the *Cnr1* gene in the mOFC as compared to low impulsive rats. **(B)** Impulsive choice (as defined by the k parameter) was positively correlated with *Cnr1* (CB_1_ cannabinoid receptor) gene expression the mOFC. **(C–E)** There were no *Cnr1* gene expression changes in the lOFC between HI-DD and LI-DD rats **(C)** or between HI-2C and LI-2C neither in the mOFC **(D)** nor in the lOFC **(E)**. The correlation is represented as the best fit regression line with dashed lines depicting the 95% confidence interval. Bar graphs represent the mean ± standard error of the mean of the fold change in gene expression. Symbols in bar graphs represent individual data points from each rat. ^∗∗^*p* < 0.01 as compared to the low impulsive group.

**FIGURE 5:**
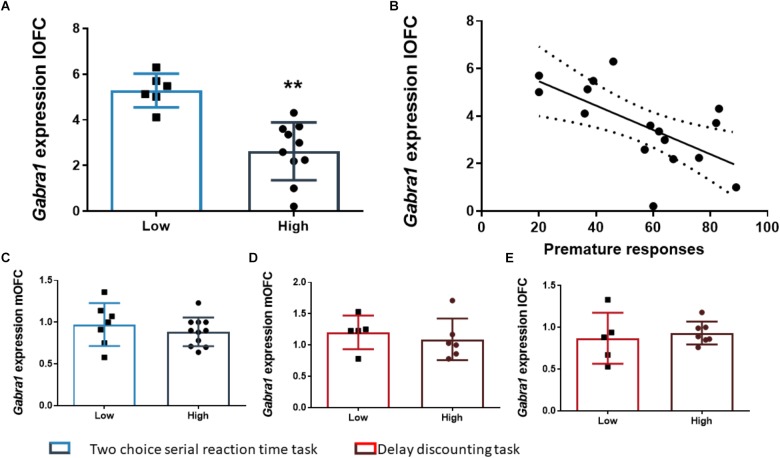
The relationship between *Gabra1* gene expression and impulsive behavior. **(A)** Animals classified as high impulsive in the 2-CSRTT showed significantly lower levels of expression of the *Gabra1* gene in the lOFC. **(B)** Impulsive action in the 2-CSRTT was negatively correlated with the gene expression of the *Gabra1* gene (which encodes the alpha 1 subunit of the GABA_A_ receptor) in the lOFC. **(C–E)** There were no *Gabra1* gene expression changes in the mOFC between HI-2C and LI-2C rats **(C)** or between HI-DD and LI-DD neither in the mOFC **(D)** nor in the lOFC **(E)**. The correlation is represented as the best fit regression line with dashed lines depicting the 95% confidence interval. Bar graphs represent the mean ± standard error of the mean. Symbols in bar graphs represent individual data points from each rat. ^∗∗^*p* < 0.01 as compared to the low impulsive group.

The analysis of the differences between the groups extracted by cluster analysis revealed that the expression of *Gabra1* in the lOFC was lower in the HI-2C as compared to LI-2C rats (*t*_15_ = 3.19; *p* < 0.01; *d* = 1.79; Figure 5A). We also found that the premature responses during the test were inversely related to the expression of *Gabra1* in the lOFC (*r* = -0.48; *p* < 0.05 uncorrected). The animals that expressed lower levels of *Gabra1* were less prone to make premature responses and hence, less impulsive (Figure 5B). There were no *Gabra1* gene expression differences between HI-2C and LI-2C in the mOFC (Figure 5C) or in *Cnr1* expression in either territory of the OFC (Figure 4D,E).

## Discussion

This study was aimed at determining if the expression of certain genes related to glutamatergic, GABAergic or endocannabinoid neurotransmission was associated to two different components of waiting impulsivity (delay-discounting and premature responding) and if there was neuroanatomical segregation between the medial and lateral divisions of the OFC in this relationship. For this purpose, we classified two separate groups of rats according to their performance in each task. A hierarchical clustering approach was chosen as the sorting strategy because, as observed from the figures, there was not a large variance between groups. We then compared the expression of selected genes related to neurotransmission in the medial and lateral orbitofrontal cortices between the resulting groups, searching for potential differences that could be specific to each variety of impulsivity.

Our results suggest that the gene expression signature of these two elements of waiting impulsivity is indeed different. We have found that, at the level of the genes studied here, the motor impulsivity component measured in the 2-CSRTT was mostly related to GABAergic gene expression in the lOFC, while the choice impulsivity assessed in the DDT was correlated with endocannabinoid gene expression in the mOFC.

The OFC has been strongly implicated in impulsiveness, goal-directed behavior and decision making-processes, although its key role in these psychological phenomena has been recently challenged (Stalnaker et al., 2015). With regard to impulsive behavior, the lesion studies that have been performed using DDT measurements of impulsive choice show conflicting results (Mobini et al., 2002; Chudasama et al., 2003; Winstanley et al., 2004; Rudebeck et al., 2006; Mar et al., 2011). The functional heterogeneity in the OFC has been suggested to be one of the reasons for such discrepancies (Mar et al., 2011; Stopper et al., 2014).

The mOFC has been proposed to be a hub where the different value signals of subjective goals are integrated (Kable and Glimcher, 2009). Indeed, mOFC-lesioned monkeys have difficulty making choices when the value of two options is close (Noonan et al., 2010) and studies with human patients have shown that mOFC lesions affect reward valuation and self-control in intertemporal choice tasks (Peters and D’Esposito, 2016). Rat lesion studies also provide evidence for a role of the mOFC in impulsive choice whereby mOFC damage increases the preference for a large but delayed reward (Mar et al., 2011). We have found that expression of *Cnr1* in the mOFC was directly related to the waiting impulsivity that is captured by the DDT. The relationship between the endocannabinoid system and the different varieties of impulsivity is complex [see Moreira et al. (2015) for an excellent review]. Some previous reports suggested that the activation of CB_1_ receptors in the OFC promote impulsive choice (Khani et al., 2015; Fatahi et al., 2018), however, these studies mainly targeted the lateral and ventral divisions of the OFC making any comparison to the present results problematic. There are also previous studies assessing the effects of systemic injections of CB_1_ receptor agonists that suggest that THC administration reduced choice impulsivity measured with the DDT (Wiskerke et al., 2011). Interestingly, another study showed no effect after treatment with a cannabinoid agonist WIN 55,512-2 (Pattij et al., 2007). It is important to note that CB_1_ receptors are mostly presynaptically localized in axon terminals, so the gene expression differences found here (arising from mRNAs in the cell bodies) could be modulating neurotransmission distally, in terminal areas such as the hippocampus, a structure that is strongly connected to the mOFC (Fettes et al., 2017). In any case, the higher levels of *Cnr1* gene expression in high impulsive animals in the mOFC may suggest that this subpopulation could be especially vulnerable to the disrupting effects of cannabinoids on those cognitive processes that depend on the normal function of the mOFC, such as reward valuation or self-control. It could also mean that, based on their differential expression of cannabinoid receptors, high impulsive individuals might reduce their impulsivity (or at least the tolerance to delay component of impulsivity) to a higher degree than low impulsive individuals, after marihuana use. This hypothesis merits further testing.

Previous studies, both in humans (Elliott et al., 2000) and monkeys (Iversen and Mishkin, 1970; Noonan et al., 2010) have shown that the lOFC is specifically required when a response previously associated with reward has to be suppressed (but see Gourley et al., 2010) and, conversely, its inactivation leads to impaired adjustment of behavior after non-rewarded actions (Dalton et al., 2016). While lesions of the lOFC have been shown to increase impulsive choice (Mar et al., 2011), to the best of our knowledge, a clear (and specific) role for the lOF in premature responding has not yet been established.

*Gabra1* expression was lower in the animals that made more premature responses in the 2-CSRTT. In forebrain pyramidal neurons, GABA_A_ receptors containing the alpha 1 subunit are mainly expressed throughout the somatodendritic region while those containing the alpha 2 subunit are mostly localized to the axon initial segment (Nusser et al., 1996; Loup et al., 1998). This differential expression of the subunit in high and low impulsive animals could translate into net differences in the cellular localization of the receptor in both populations and this might have implications for how inhibitory signals are integrated by the cortical pyramidal neurons where these receptors are expressed. There are other previous studies that have involved the GABAergic system in impulsive action. For example, Jupp et al. (2013) found that GABA_A_ binding in the anterior cingulate cortex was negatively correlated with premature responding in the 5-CSRTT and Caprioli and co-workers established a role of the GABA synthesis enzyme GAD (glutamic acid decarboxylase) within the nucleus accumbens core in premature responding (Caprioli et al., 2014) In addition, GAD inhibition in the medial prefrontal cortex impaired impulse control measured in the 5-CSRTT (Paine et al., 2015).

## Conclusion

In conclusion, we here provide the first evidence for a dissociation between the medial and lateral division of the OFC in impulsive action and impulsive choice and suggest that CB_1_ receptors in the mOFC are positively coupled to the expression of impulsive choice while GABA_A_ receptors in the lOFC are markers of impulsive action. Functional studies interfering with or augmenting the expression of these genes must now be conducted in order to ascertain if there is a causal relationship between the gene transcription variations here reported and the different varieties of waiting impulsivity that we have studied in this work.

## Author Contributions

AH-M conceived the research and carried out the initial experiments. MU performed the experiments and analyzed the data. AC performed the impulsive action behavioral experiments and qPCRs. DR-M performed qPCR experiments and helped with data analysis. SP-R helped with the qPCR experiments. JO assisted with data analysis and interpretation. AH-M, EA, and MU wrote the manuscript with the feedback of the rest of the authors.

## Conflict of Interest Statement

The authors declare that the research was conducted in the absence of any commercial or financial relationships that could be construed as a potential conflict of interest.
