# Supplementary material for: Impulsive Action and Impulsive Choice Are Differentially Associated With Gene Expression Variations of the GABAA Receptor Alfa 1 Subunit and the CB1 Receptor in the Lateral and Medial Orbitofrontal Cortices
Source: Front Behav Neurosci. 2019 Feb 20;13:22. doi: 10.3389/fnbeh.2019.00022 (PMC6391359; doi:10.3389/fnbeh.2019.00022)
Supplement: Supplementary file 1 [file Data_Sheet_1.docx]

**Supplementary Information:**

**Alternative analyses of the delay-discounting task with area under the curve (AUC) and normalized AUC**

**There is a debate in the preclinical literature on impulsivity about the best way to analyze impulsivity using delay-discounting curves (the two debated options are curve-fitting approaches or to compute the area under the delay-discounting curve). In addition to extracting the k parameter (see Methods section) we have also computed the AUC (normalized and non-normalized). When using these AUCs as input variables, the clustering approach was inadequate as it created two groups with a very unequal number of animals, even after removing two outliers (see SI Fig.1). In spite of this, the correlation obtained for *Cnr1* and the k parameter had an equivalent correlation between both the AUC (Magnard et al., 2018) and the normalized AUC (Myerson et al., 2001), with or without two identified outliers, and *Cnr1,* supporting the robustness of our results.**


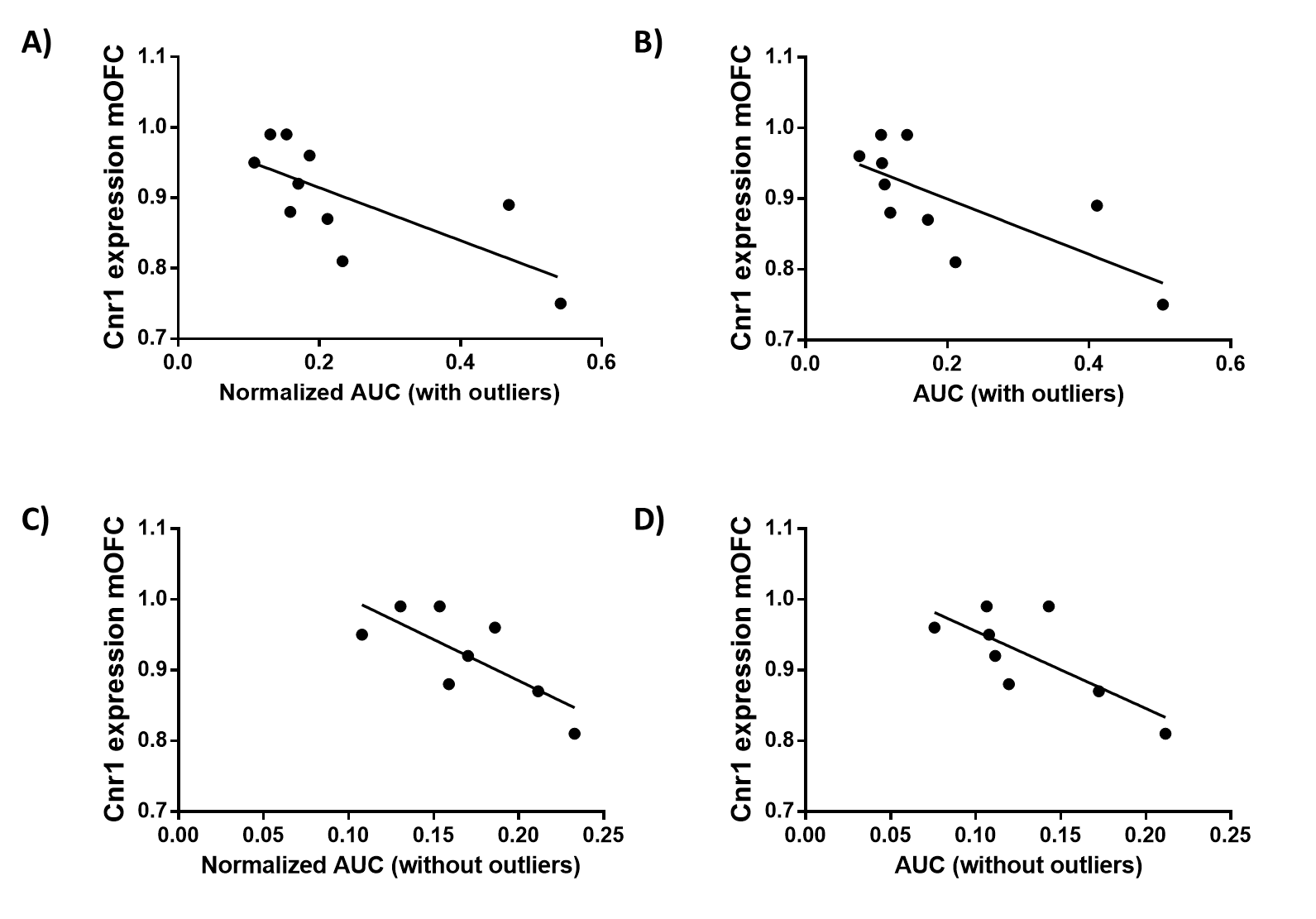


**SI. Fig 1. The relationship between *Cnr1* gene expression and impulsive behavior measured with the AUC methods (alternative analysis)**

**Impulsive choice was positively correlated with impulsivity measured by both measures of AUC, with or without outliers (a smaller AUC indicates a faster switch in the preference in favor of the immediate lever i.e. higher impulsivity) A) Impulsive choice (as defined by the normalized AUC, without removing outliers) was positively correlated with *Cnr1* (CB_1_ cannabinoid receptor) gene expression in the mOFC (Kendall’s τ=-0.584; p=0.02). B) Impulsive choice (as defined by the AUC, without removing the outliers) was positively correlated with *Cnr1* (CB_1_ cannabinoid receptor) gene expression the mOFC (Kendall’s τ-0.629=; p=0.012). C) Impulsive choice (as defined by the normalized AUC without the two outliers) was positively correlated with *Cnr1* (CB_1_ cannabinoid receptor) gene expression the mOFC (Pearson’s r=-0.747; p=0.033). D) Impulsive choice (as defined by the AUC without the outliers) was positively correlated with *Cnr1* (CB_1_ cannabinoid receptor) gene expression in the mOFC (Pearson’s r=-0.740; p=0.036).**

**SI Table 1. Primer sets used for RT-qPCR amplification.**

| **Gene** | **Description** | **Forward primer** | **Reverse primer** |
| --- | --- | --- | --- |
| *Grin1* | glutamate ionotropic receptor NMDA type subunit 1 | AACCTGCAGAACCGCAAG | GCTTGATGAGCAGGTCTATGC |
| *Grin2a* | glutamate ionotropic receptor NMDA type subunit 2A | TGTGAAGAAATGCTGCAAGG | GAACGCTCCTCATTGATGGT |
| *Gria1* | glutamate ionotropic receptor AMPA type subunit 1 | AGAGGCTGGTGGTGGTTGACT | ACCCTGGTATGGTCTCGGGA |
| *Gria2* | glutamate ionotropic receptor AMPA type subunit 2 | GGCGTGTAATCCTGGACTGT | ACACCAGGGAATCGTCGTAG |
| *Gabrg2* | gamma-aminobutyric acid type A receptor gamma 2 subunit | CGGAAACCAAGCAAGGATAA | ACAGTCCTTGCCATCCAAAC |
| *Gabrd* | gamma-aminobutyric acid type A receptor delta subunit | GCTGGACCTGGAGAGCTATG | CCGAAGCTGGAAGTGTAAGC |
| *Gabra1* | gamma-aminobutyric acid type A receptor alpha 1 subunit | TTGACTGTGAGAGCCGAATG | AAACGTGACCCATCTTCTGC |
| *Gabra2* | gamma-aminobutyric acid type A receptor alpha 2 subunit | CCATGCACTTGGAGGACTT | ACTGGCCCAGCAAATCATAC |
| *Cnr1* | cannabinoid receptor 1 | GTCGATCCTAGATGGCCTTGC | GTCATTCGAGCCCACGTAGAG |
| *Dagla* | diacylglycerol lipase, alpha | CTTTGCTGAATTTTTCCGTGACC | TTGTTTGCCTCATCCAGCAC |
| *Mgll* | monoacylglycerol lipase | CTACCTGCTCATGGAATC | GACACCCACGTATTTATTTC |
| *Napepld* | *N*-acyl phosphatidylethanolamine phospholipase D | AGATATGGACTCAAGAGTGAAGACTTC | TCCTCAAAGGCTTTGTCATCGG |
| *Faah* | fatty acid amide hydrolase | GTTACAGAGTGGAGAGCTGTCC | GTCTCACAGTCGGTCAGATAGG |
| *Gapdh* | glyceraldehyde-3-phosphate dehydrogenase | TCCCTGTTCTAGAGACAG | CCACTTTGTCACAAGAGA |

**SI Table 2. Uncorrected p-values for all the t-tests performed. Red cells show significant values and cells with border contain the values that are significant after the FDR correction.**

| **Statistical aproach** | **Clustering aproach** | | | | **Correlational aproach** | | | |
| --- | --- | --- | --- | --- | --- | --- | --- | --- |
|  | DDT | | 2-CSRTT | | DDT | | 2-CSRTT | |
|  | lOFC | mOFC | lOFC | mOFC | lOFC | mOFC | lOFC | mOFC |
| *Grin1* | 0,972 | 0,814 | 1,000 | 0,424 | 0,683 | 0,842 | 0,189 | 0,336 |
| *Grin2a* | 0,947 | 0,374 | 0,069 | 0,549 | 0,945 | 0,805 | 0,449 | 0,484 |
| *Gria1* | 0,763 | 0,263 | 0,035 | 0,792 | 0,913 | 0,040 | 0,351 | 0,848 |
| *Gria2* | 0,711 | 0,792 | 0,157 | 0,915 | 0,732 | 0,938 | 0,518 | 0,672 |
| *Gabrg2* | 0,485 | 0,933 | 0,100 | 0,981 | 0,845 | 0,609 | 0,231 | 0,784 |
| *Gabrd* | 0,338 | 0,933 | 0,660 | 0,905 | 0,177 | 0,815 | 0,902 | 0,646 |
| *Gabra1* | 0,636 | 0,559 | **0,006** | 0,394 | 0,889 | 0,223 | 0,0498 | 0,585 |
| *Gabra2* | 0,836 | 0,886 | 0,558 | 0,832 | 0,835 | 0,820 | 0,811 | 0,744 |
| *Cnr1* | 0,421 | **0,003** | 0,936 | 0,609 | 0,266 | 0,009 | 0,669 | 0,324 |
| *Dagla* | 0,940 | 0,153 | 0,967 | 0,964 | 0,430 | 0,178 | 0,476 | 0,595 |
| *Mgll* | 0,863 | 0,927 | 0,202 | 0,536 | 0,956 | 0,896 | 0,470 | 0,804 |
| *Napepld* | 0,621 | 0,111 | 0,065 | 0,126 | 0,337 | 0,048 | 0,052 | 0,640 |
| *Faah* | 1,000 | 0,063 | 0,425 | 0,724 | 0,835 | 0,436 | 0,955 | 0,515 |
